# Tools to Reduce Low-Value Care: Lessons From COVID-19 Pandemic: Comment on "Key Factors that Promote Low-Value Care: Views of Experts From the United States, Canada, and the Netherlands"

**DOI:** 10.34172/ijhpm.2022.6887

**Published:** 2022-04-12

**Authors:** Luis Corral-Gudino

**Affiliations:** ^1^Medicine, Dermatology and Toxicology Department, Universidad de Valladolid, Valladolid, Spain.; ^2^Internal Medicine Department, Hospital Universitario Río Hortega, Valladolid, Spain.

**Keywords:** Low-Value Care, De-Implementation, Medical Overuse, Overtreatment, Overdiagnosis, COVID-19

## Abstract

Based on a summary of interviews with 18 experts, Verkerk et al defined the seven key factors that promoted low-value care, which included system, social, and knowledge factors. During the ongoing coronavirus disease 2019 (COVID-19) pandemic, these key factors have been influential due to the uncertainty of the disease at the beginning of the pandemic. Globally, several measures have been implemented to reduce low-value care practices and promote high-value care for COVID-19 patients. From huge multicenter, non-industry sponsored or multiplatform trials, to the use of social networks sites is an indispensable and effective way to disseminate medical information. Thanks to these measures, we have transformed a scenario of ignorance into an evidence-based medical scenario in less than a year. Verkerk and colleagues’ proposed key factors are an excellent framework for characterizing and highlighting the lessons that can be learnt from how we have fought against the pandemic and low-value practices.

 In healthcare systems, the patients’ needs, expectations, and preferences should guide the care. However, these are dependent on the skills and knowledge of the health professionals, the availability of financial and material resources, the commercial interests, or the cultural, social, or ethical values. This complexity allows the persistence of low-value care practices. These practices, which do not improve patients’ health nor add value to care, would be unthinkable in other areas.

 Verkerk et al^1^ constructed a framework which identified the key factors that promoted low-value care. To address this, they interviewed experts from three different countries, each with a different healthcare payment structure, industry, or malpractice policy, which included predominantly privately financed (the United States) and predominantly publicly financed healthcare systems (Canada and the Netherlands).

 The most remarkable contribution of the manuscript was the identification and characterization of seven factors that promoted low-value care. The factors were classified in three groups: (1) system factors, which include the fee-for-service system, pharmaceutical and medical device industry, and fear of malpractice litigation; (2) knowledge factors, which include medical education and biased evidence and knowledge; and (3) social factors, which include the “more is better” public and medical culture (Figure 1). Although the authors did not identify the same factors as the seminar paper of Saine et al,^2^ most of them are superimposable and both the frameworks seem to be complementary.

**Figure 1:**
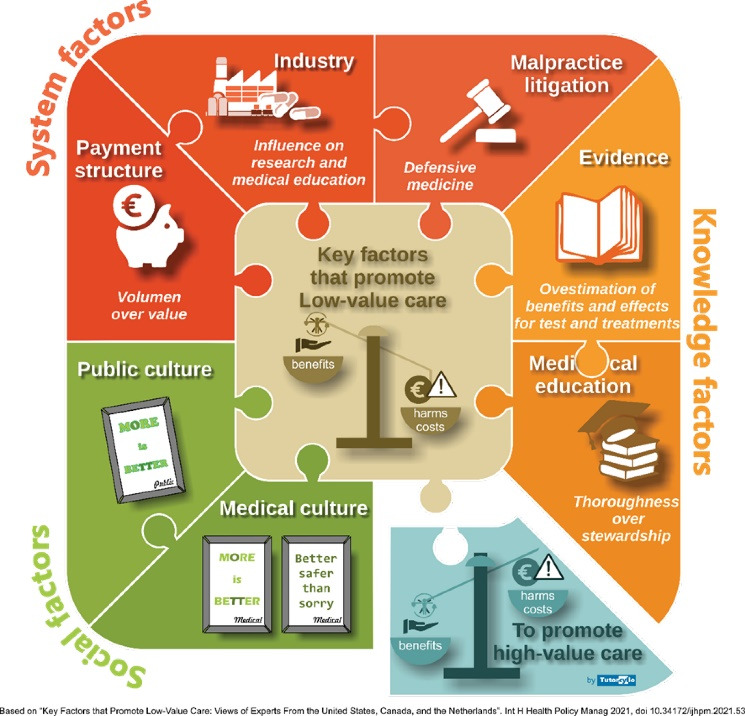
Seven Key Factors That Promoted Low-Value Care (Verkerk et al Framework).

 The authors aimed to deepen the knowledge of the factors that promoted and maintained low-value care. The recent coronavirus disease 2019 (COVID-19) pandemic changed patient care and lead to an increase in low-value care, at least during the first few months. The analysis of these changes through the seven key factors proposed by Verkerk et al could help us highlight what the pandemic has taught us regarding low-value care.

## The Impact of the COVID-19 Pandemic on Increased Low-Value Care

 In 2020, all healthcare systems and related professionals globally went through a tremendously demanding test during the COVID-19 pandemic. The healthcare systems were confronted simultaneously with the uncertainty of an unknown disease with severe presentation and rapid transmission, the lack of useful treatments against the disease, the urgent need for drug repositioning, the development of protocols and guidelines in a state of constant change, the avalanche of manuscripts peer-reviewed or not, and the misinformation related to COVID-19, which amplified “good” and “bad” science with the widespread use of social networks.

 During the first months of 2020, these circumstances created a perfect storm to replace evidence-based medicine (EBM) with Stupor-based Medicine^3^ and to see the flourishing of low-value care practices, from the overuse of antiviral treatments without evidence against COVID-19 or the use of antibiotics without indication for a viral disease, to the underuse of immunosuppressive drugs, such as corticosteroids, based on previous experiences with other diseases, such as influenza.

 However, in less than half a year, EBM knowledge regained its place, and most of the low-value care used at the beginning of the pandemic was reduced or even de-implemented. For example: Hydroxychloroquine was used in the United States in more than half the patients in March 2020 and in only 0.8% of patients in August 2020.^4^ In Spain, corticoisteroids^5^ were used in 51.6% of the severe patients in March 2020 and in 85.7% in September 2020, while the inappropriate use of antibiotics^6^ reached 35% in March 2020 and dropped to 28% in April 2020. How was it possible to reduce all these low-value practices within a limited period?

## System Factors

 Changes in industry and defensive medicine, two of the three key system factors, have been the main driving forces behind the reduction of low-value care during the COVID-19 pandemic.

 While vaccines and new drugs specific for COVID-19 have been developed by the pharmaceutical industry, multiple “old” treatments have been repositioned for their use against COVID-19 worldwide. For months, cases series or retrospective studies with conflicting results and inadequate designs were the only source of “evidence.” This led to the development of local protocols, most of which were clearly inadequate according to current knowledge. However, a few key trials published from the summer of 2020 were able to achieve the implementation or de-implementation of the different drugs and homogenize COVID guidelines according to real EBM. The questions are: who were the researchers who put forth the initiatives to test the drugs used against COVID-19 without funding of the pharmaceutical industry, and how the results of these key trials were introduced in clinical practice so quickly?

 An initiative, known as RECOVERY^7^ has entirely changed our way of conceiving the development of clinical trials not funded by the pharmaceutical industry. This trial integrated tools from clinical trials, such as randomization, into routine clinical care with the provision of an adaptive clinical trial design. Most of the safety assessments and efficacy of the different repositioned drugs for COVID-19 were derived from this study. It is true that there were a few more international initiatives, such as the World Health Organization’s (WHO’s) SOLIDARITY trial,^8^ and hundreds of small local or regional initiatives. However, none of them had the importance of this, both for the originality of its design and the power of its results. This type of mega study not linked to the pharmaceutical industry should be applied to other pathologies.

 The other systemic factor modified during the pandemic was the publication of official documents to support the rational use of resources. The overwhelming magnitude of the health crisis at the beginning forced medical societies to take a firm stance on the care that could or could not be provided. For example, the Italian Society of Anesthesia Analgesia Resuscitation and Intensive Care published a document^9^ for healthcare professionals that could be used as a reference for medico-legal assessment in cases of dispute. The publication of such documents for the different wisely recommended choices could reassure the clinicians against the fear of being sued and reduce the practice of defensive medicine.

## Knowledge Factors

 Medical education and evidence were the two knowledge factors described in Verkerk and colleagues’ framework. From the beginning, different organizations developed sets of COVID-19 recommendations based on the usual choice of campaigns to reduce low-value practices.^10^ Regrettably, the only publication of these recommendations did not guarantee their implementation. The use of multicomponent interventions targeting clinicians proved to be more effective than the dissemination of recommendations to reduce the use of low-value health services.^11^

 In a situation where there was a need for urgent diffusion of clinical evidence, the promotion of medical conferences was an intervention of utmost importance to disseminate these recommendations. Usually, medical conferences are expensive and requires a need to travel and take days off work. In addition, the funding of medical conferences depends largely on the pharmaceutical industry. Due to the travel restrictions imposed for the COVID-19 pandemic and the urgent need to develop COVID-19 monographic conferences, we witnessed the accelerated development of online or online plus local (hybrid) medical conferences. These kinds of conferences brought at least four important advantages: (1) the conference price was reduced dramatically, and was free for the remote version, (2) most conferences were organized and funded by medical societies or government agencies. Hence, they eliminated the need for funding from the pharmaceutical industry and thus were able to avoid commercial interests, (3) most sessions were recorded, which allowed the conference attendees to watch the sessions on demand at any time after the live session, and (4) the recorded sessions created a repository of information that could be shared and used by a larger audience, instead of just the congress attendees. The possibility of online attendance with a much lower registration price, the creation of an online repository of the recorded sessions available to the medical community, and the development of the role of government agencies as main funders for medical conferences are changes that should remain. This is to ensure that medical congresses do not become ivory towers to which individuals can go only when invited by the industry or by spending parts of their research funds. In recent years, medical education has moved from a scenario where education is reduced to faculty classrooms to a much broader scenario where the web plays a leading role. The recorded medical sessions could be part of the learning tools used to promote autonomous learning.

 Another interesting finding during COVID-19 was that multi-country collaborations shared data by creating public repositories to present comparable indicators of COVID-19. In addition, the development of multiplatform trials was a significant advancement. One of the most remarkable examples was the collaboration between the Randomised, Embedded, Multi-factorial, Adaptive Platform Trial for Community-Acquired Pneumonia (REMAP-CAP), Accelerating COVID-19 Therapeutic Interventions and Vaccines (ACTIV-4), and Antithrombotic Therapy to Ameliorate Complications of COVID-19 (ATTACC).^12^ These three platforms were able to homogenize their clinical trial protocols and run their three clinical trials simultaneously to summarize their outcomes, leading to more evidence-based data. The generosity and extra work involved in such a collaboration is a powerful weapon to fight low-value care.

 However, not everything has been positive in relation to scientific evidence with evolution of the pandemic. Quite to the contrary, some authors have demonstrated that the pandemic has reduced quality standards of research, with the publication of articles with lower methodological quality scores and the dissemination of non-peer reviewed manuscripts.^13^

## Social Factors

 Social and medical culture were the two social factors identified by Verkerk et al. Culture is defined by the Cambridge dictionary as “the way of life, especially the general customs and beliefs, of a particular group of people at a particular time.” Social networks are a way to export this way of life. COVID-19 generated an avalanche of over-abundant information, or an infodemic. The Internet has become the easiest and fastest source of health information. However, social media is equally efficient in spreading both information and misinformation. Therefore, during the pandemic, the propagation of misleading information online notably increased low-value care. To fight against misinformation, most healthcare organizations and physicians had to embrace an active and public role in social networks. A set of actions for the smart use of social networks was proposed as a response to this infodemic.^14^ This response required swift, coordinated, and regular actions on social media, coming from multiple sectors, such as healthcare organizations, health professionals, and patients. As the body of information grew during the pandemic, the rapid dissemination of changing updates to communities and individuals through social networks was of paramount importance in implementing good practices and reducing bad ones. Health educators should act as influencers on social media, with attractive, easily readable, and updated content. An additional effort should be made not only to disseminate good science on social media but also empower health professionals and citizens to be able to separate the wheat from the chaff when using social media and demand trusted evidence-based information.

 Social factors could not be reduced to social networks or public and medical culture. Internal mechanism, as biases (cognitive or emotional) or imperatives,^15^ play a key role in the promotion or reduction of the use of low-value care practices. During the COVID-19 pandemic, the role of psychological factors in decision-making have been enhanced due the uncertainty generated by facing a disease for which initially we had neither evidence nor guidelines.

## What the COVID-19 Pandemic Can Teach us About the Reduction of Low-Value Care Practices

**Figure 2:**
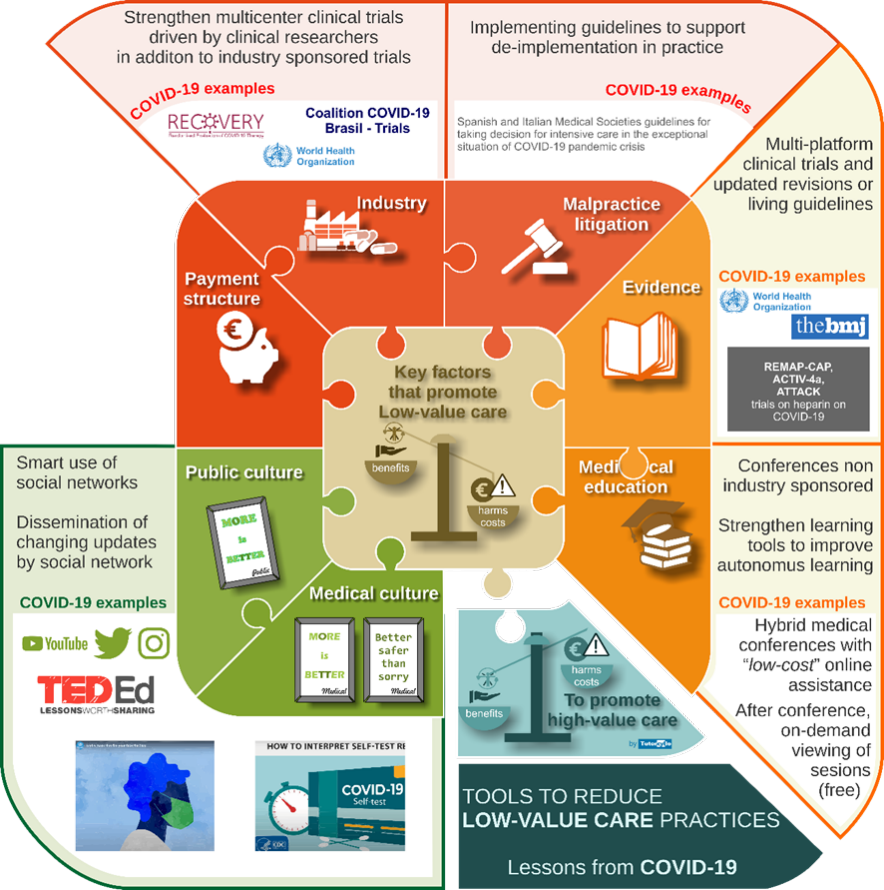
Changes in the Different Key Factors That Promoted Low-Value Care During the COVID-19 Pandemic.

 The COVID-19 pandemic changed the way we understand healthcare. Never before have low-value care practices spread so quickly globally, as at the beginning of the pandemic. Undoubtedly, the use of this kind of practice was reduced rapidly and widely in the following months. The seven key factors that promoted low-value care identified by Verkerk et al are a magnificent tool to analyze the changes in healthcare and EBM that emerged during this period and to be able to understand the lessons learnt in response to the pandemic. The changes in the different key factors during the COVID-19 pandemic are summarized in Figure 2.

## Ethical issues

 Not applicable.

## Competing interests

 Author declares that he has no competing interests.

## Author’s contribution

 LCG is the single author of the paper.
